# GeneAgent: self-verification language agent for gene-set analysis using domain databases

**DOI:** 10.1038/s41592-025-02748-6

**Published:** 2025-07-28

**Authors:** Zhizheng Wang, Qiao Jin, Chih-Hsuan Wei, Shubo Tian, Po-Ting Lai, Qingqing Zhu, Chi-Ping Day, Christina Ross, Robert Leaman, Zhiyong Lu

**Affiliations:** 1 Division of Intramural Research, National Library of Medicine, National Institutes of Health, Bethesda, MD USA; 2 Cancer Data Science Laboratory, Center for Cancer Research, National Cancer Institute, National Institutes of Health, Bethesda, MD USA

**Keywords:** Machine learning, Software, Genomics

## Abstract

Gene-set analysis seeks to identify the biological mechanisms underlying groups of genes with shared functions. Large language models (LLMs) have recently shown promise in generating functional descriptions for input gene sets but may produce factually incorrect statements, commonly referred to as hallucinations in LLMs. Here we present GeneAgent, an LLM-based AI agent for gene-set analysis that reduces hallucinations by autonomously interacting with biological databases to verify its own output. Evaluation of 1,106 gene sets collected from different sources demonstrates that GeneAgent is consistently more accurate than GPT-4 by a significant margin. We further applied GeneAgent to seven novel gene sets derived from mouse B2905 melanoma cell lines. Expert review confirmed that GeneAgent produces more relevant and comprehensive functional descriptions than GPT-4, providing valuable insights into gene functions and expediting knowledge discovery.

## Abstract (web summary)

GeneAgent is a language agent using large language models and self-verification to improve gene-set function annotation.

## Main

Gene-set analysis allows researchers to explore groups of genes that likely act together in specific biological processes or molecular functions^1–4^. This approach builds on the extensive results of mRNA expression experiments and proteomics studies, which have identified differentially expressed sets of genes and proteins^5,6^. Utilizing the assumption that these genes work together, many computational methods use Gene Ontology (GO) annotations to elucidate the underlying biological mechanisms^7,8^.

Gene-set enrichment analysis (GSEA), a cornerstone of functional genomics, measures the overrepresentation or underrepresentation of biological functions associated with a set of genes or proteins^9–12^. GSEA typically compares gene clusters against predefined categories in manually curated databases, such as GO^13^ and the Molecular Signatures Database (MSigDB)^14,15^, using rank-based metrics. However, gene sets exhibiting strong enrichment in the existing databases have often been well analyzed by previous research. Therefore, an increasing number of recent studies focus on gene sets that only marginally overlap with known functions^16^.

LLMs have emerged as promising tools for gene-set analysis due to their powerful reasoning capability and rich modeling of biological context^17,18^. LLM agents leveraging instruction learning^19,20^ and multi-agent conversation^21^ and integrating with external resources show particular promise. Jin et al.^20^ presented GeneGPT to answer genomics questions by teaching LLMs to use the web tools. Wu et al.^21^ developed AutoGen to build applications of various complexities and LLM capacities potentially for genomic question answering. Hu et al.^16^ evaluated the performance of five LLMs in gene-set analysis by designing a set of prompts to identify the functions of genes within a given gene set. Another work using standard LLMs, SPINDOCTOR^22^, summarizes multiple biological process names from various resources given a gene set.

However, previous studies did not often investigate hallucinations, where LLMs generate plausible yet fallacious contents, a common problem in general-purpose LLMs. This potential for fabricated and inaccurate results poses a challenge for creating a reliable framework to accurately generate biological process names for gene sets and hinders the objective interpretability of gene functions.

**Fig. 1:**
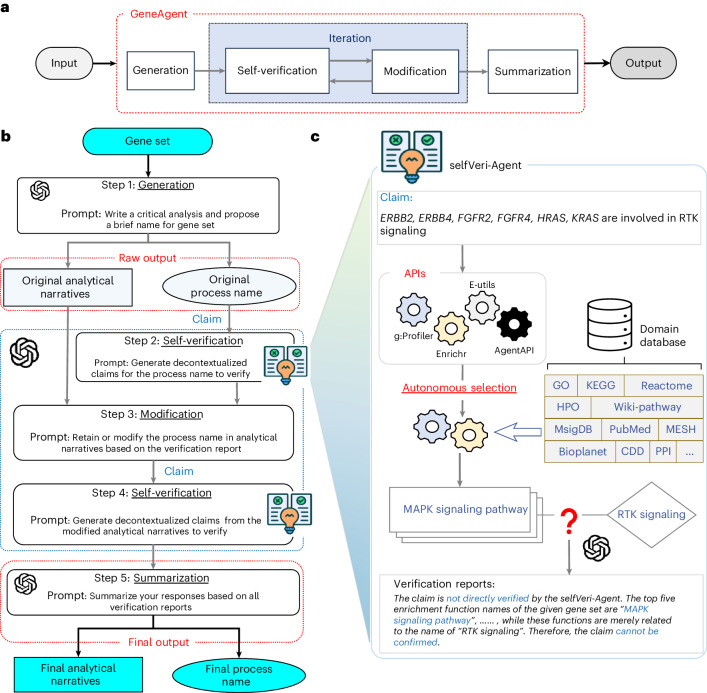
Framework of GeneAgent for gene-set analysis. **a**, An overview of GeneAgent, containing generation, self-verification, modification and summarization. The self-verification module is iterated through the modification module. **b**, The workflow of GeneAgent in the gene-set analysis. When obtaining the input gene set, the Generation step creates the raw outputs containing the process name and the analytical narratives using LLMs configured with the engineered prompts proposed by Hu et al.^16^. The Self-verification step identifies the hallucinations in the raw output based on the claims generated from the process name or the analytical narrative. The Modification step corrects hallucinations in the verification of the process name and activates the verification of analytical narratives. The Summarization step produces the final output containing the updated process name and the analytical narratives for the input gene set. Detailed prompts are provided in Supplementary Document 1. **c**, The demonstration of selfVeri-Agent with a claim example of ‘ERBB2 (erb-b2 receptor tyrosine kinase 2), ERBB4 (erb-b2 receptor tyrosine kinase 4), FGFR2 (fibroblast growth factor receptor 2), FGFR4 (fibroblast growth factor receptor 4), HRAS (HRas proto-oncogene, GTPase), KRAS (KRAS proto-oncogene, GTPase) is involved in RTK signaling’ generated from the process name. A complete example is shown in Supplementary Table 2.

To overcome these challenges, we developed GeneAgent, a language agent built upon GPT-4 to automatically interact with domain-specific databases to annotate functions for gene sets. GeneAgent generates interpretable and contextually accurate biological process names for user-provided gene sets, either aligning with significant enrichment analyses or introducing novel terms. At the core of GeneAgent’s functionality is a four-stage pipeline centered on self-verification (Fig. 1a). This mechanism autonomously interacts with various expert-curated biological databases through Web APIs. By utilizing relevant domain-specific information, GeneAgent performs fact verification and provides objective evidence to support or refute the raw LLM output, reducing hallucinations and enabling reliable, evidence-based insights into gene function.

We evaluated GeneAgent on gene sets from three distinct sources: literature curation (GO), proteomics analyses (nested systems in tumors (NeST) system of human cancer proteins^6^) and molecular functions (MSigDB). All datasets were released after 2023, while the version of GPT-4 used in GeneAgent has training data up to September 2021 (Methods). GeneAgent significantly outperformed recent LLMs using the prompts proposed by Hu et al.^16^ (Supplementary Table 1). Compared with the gene function synopsis used by SPINDOCTOR, GeneAgent provides more informative gene summaries for LLMs to generate relevant biological terms. These improvements are primarily due to GeneAgent’s reduction in hallucinations, thereby enhancing accuracy.

In a real-world application, we tested GeneAgent on seven novel gene sets derived from mouse B2905 melanoma cell lines. GeneAgent not only achieved better performance compared to GPT-4 but also offers valuable insights into novel gene functionalities, facilitating knowledge discovery. This use case also shows GeneAgent is robust across multiple species.

## Results

### GeneAgent workflow

GeneAgent enhances the accuracy of gene-set analysis by minimizing instances of hallucinations through an advanced self-verification feature. This feature allows GeneAgent to autonomously interact with domain-specific databases and refine the raw output from the LLM (Methods and Box 1). A complete example is shown in Supplementary Table 2.

Specifically, the input to GeneAgent is a gene set provided by the user (Fig. 1b), which GeneAgent processes to create the raw output, containing a preliminary process name and several paragraphs analyzing the functions of the input genes. GeneAgent then activates the self-verification agent (selfVeri-Agent; Fig. 1c) to verify the process name and the associated analyses. During verification, GeneAgent identifies any hallucinations by extracting claims from the raw output and comparing these against curated knowledge in domain-specific databases. The gene symbols within the claims are used to query the Web APIs of the backend databases, retrieving the associated manually curated functions.

Based on the curated gene functions, the selfVeri-Agent compiles a verification report that categorizes each claim as ‘supported’, ‘partially supported’ or ‘refuted’. The selfVeri-Agent first verifies the ‘process name’ before examining the modified ‘analytical narratives’ to ensure the process name is verified twice (Fig. 1b). Finally, GeneAgent consolidates all intermediate verification reports to produce the final updated outputs. This cascading structure improves the traditional step-by-step chain-of-thought reasoning process^23^, enabling autonomous verification of the inference process^24^. A comparison with the GPT-4 chain-of-thought process shows improved performance (Supplementary Fig. 1). GeneAgent incorporates domain knowledge from 18 biomedical databases via four Web APIs. To prevent data leakage, we implemented a masking strategy that ensures no database is used to verify its own gene sets during self-verification (Methods).

Box 1 Glossary **Ground truth**

The gold-standard functional name of input gene sets.

**Raw output**

The preliminary contents generated by LLMs needed to be verified.

**Claim**

The affirmative sentence related to the raw output of an LLM.

**Curated function**

The functions of genes that are manually curated in databases.

**Verification report**

The evidence from backend databases to verify claims.

**Gene synopsis**

The gene descriptions curated in databases or verification reports.

### GeneAgent outperforms the standard GPT-4 in benchmarks

We evaluated the performance of GeneAgent in generating relevant biological process names for a given gene set, compared with GPT-4. To ensure a fair comparison, we applied the prompt proposed by Hu et al.^16^ on the same GPT-4 used in GeneAgent but did not apply the self-verification, denoting this setup as ‘GPT-4 (Hu et al.)’. The gene sets analyzed range in size from 3 to 456, with an average of 50.67 (Table 1).

**Table 1:** The statistics for gene sets used in our study

Gene sets used for empirical evaluation.						
Dataset	No. of sets	No. of genes	Average genes	Average word count in ground truth	Resource	Released date
GO	1,000	3 to 456	48.32	4.704	Literal curation	Nov 2023
NeST	50	5 to 323	18.96	2.214	Proteomics analysis	Apr 2024
MSigDB	56	4 to 200	112.00	2.980	Molecular function	May 2023
All	1,106	3 to 456	50.67	4.500		

Seven novel gene sets used in our real-world evaluation case study.			
ID	No. of genes	Ground truth	Resource
mmu05171 (HA-R)	36	Coronavirus disease 2019	Preclinical study of melanoma^32^ (mouse B2905 melanoma cell lines)
mmu03010 (HA-R)	35	Ribosome	
mmu03010 (HA-S)	49	Ribosome	
mmu05171 (HA-S)	47	Coronavirus disease 2019	
mmu04015 (HA-S)	27	Rap1 signaling pathway	
mmu05100 (HA-S)	19	Bacterial invasion of epithelial cells	
mmu05022 (LA-S)	24	Pathways of neurodegeneration—multiple diseases	

**Fig. 2:**
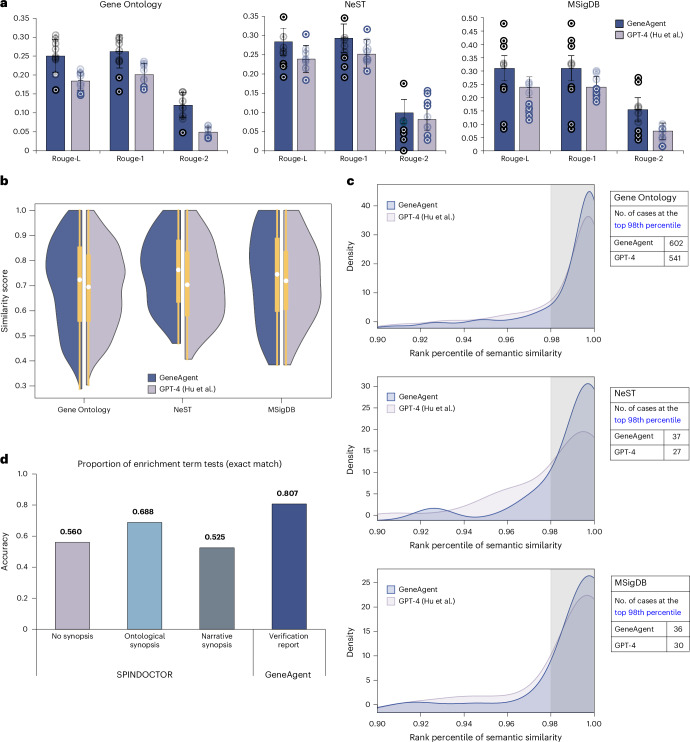
The biological process names generated by GeneAgent are more similar with their ground truth than those produced by GPT-4 using the prompts proposed by Hu et al.^16^. **a**, The ROUGE scores of GeneAgent and GPT-4 were evaluated across three datasets: 1,000 gene sets from GO, 50 from NeST and 56 from MSigDB. The s.d. for each bar was calculated using nine-fold cross-validation based on batch size (bs) sampling, with bs = 200 for GO and bs = 20 for both NeST and MSigDB. The ROUGE score for each batch size is presented in the figure. The central value of the error bars represents the mean score across all samples. The results are presented as the mean ± s.d. **b**, Distribution of similarity scores obtained by GeneAgent and GPT-4 in three datasets. The total number of gene sets used for the statistics is 1,000 (GO), 50 (NeST) and 56 (MSigDB). The middle points represent the mean values; bounds of the inner boxes of each violin plot represent the upper and lower percentiles; and whiskers represent the minimum and maximum points within all data samples. The statistically significant *P* value is 3.1 × 10^−5^ for 1,106 evaluated gene sets, which is calculated by a one-tailed *t*-test with 95% confidence intervals. The results are reported as the mean ± s.d., calculated from all similarity scores obtained by GeneAgent or GPT-4. **c**, The percentile distribution of semantic similarity between generated names and their ground truths was assessed across all candidate background terms. This background set comprises 12,320 terms, including 12,214 GO biological process terms used by Hu et al.^16^ and all available annotated terms in NeST (50) and MSigDB (56). The plot illustrates the distribution of gene sets within the top 90th percentile. The caption values represent the number of gene sets in GeneAgent and GPT-4 that fall within the top 98th percentile (that is, shadings shown in the figures). **d**, The accuracy of tested terms that exactly match the significant enrichment terms obtained by GSEA. Each value on the bar is calculated by the proportion of exact matched terms within all terms tested by the GeneAgent or SPINDOCTOR. Source data

First, we evaluate ROUGE scores (recall-oriented understudy for gisting evaluation)^25^ between the generated names and their ground truths, specifically ROUGE-L (longest common subsequence), ROUGE-1 (1-gram) and ROUGE-2 (2-gram) scores (Methods). GeneAgent demonstrated better alignment with ground-truth token sequences than GPT-4 (Hu et al.^16^; Fig. 2a). Across 1,106 gene sets with an average ground-truth length of 4.50 words (Table 1), GeneAgent achieved significant improvements over GPT-4. Notably, in the MSigDB dataset, GeneAgent improved the ROUGE-L scores from 0.239 ± 0.038 to 0.310 ± 0.047 compared to GPT-4. The ROUGE-1 scores matched ROUGE-L, and the ROUGE-2 scores also improved, from 0.074 ± 0.030 to 0.155 ± 0.044.

**Extended Data Fig. 1:**
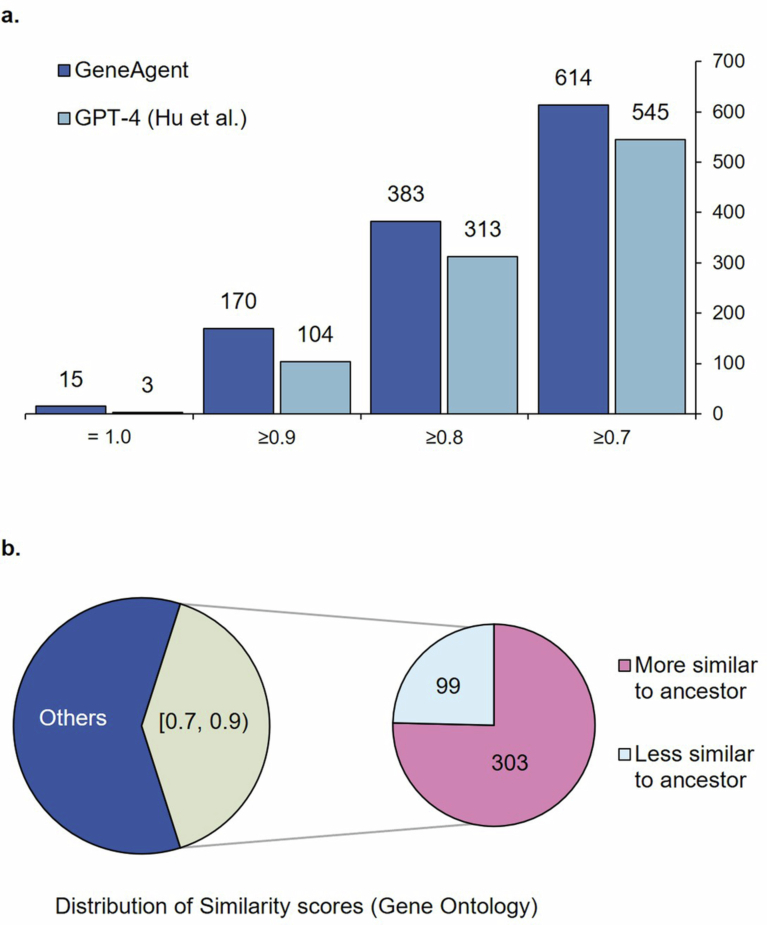
The details of similarity scores distributed in the evaluated gene sets. **a**. Statistic of gene sets at different similarity levels obtained by GeneAgent and GPT-4 (Hu et al.). The minimum cutoff value of similarity score is set to 0.7. **b**. Distribution of gene sets in the Gene Ontology dataset (n = 1000) that exhibit a higher similarity score to the ancestors of ground truth than the ground truth itself. The similarity scores of the gene sets included in the analysis range from 0.7 to 0.9 when compared with the ground truth. Source data

Next, we measured the semantic similarity between the names generated and their ground truths using MedCPT^26^, a state-of-the-art biomedical text encoder (Methods). GeneAgent achieved consistently higher average similarity scores across three datasets: 0.705 ± 0.174, 0.761 ± 0.140 and 0.736 ± 0.184, compared to GPT-4’s scores of 0.689 ± 0.157, 0.708 ± 0.145 and 0.722 ± 0.157, respectively (Fig. 2b). Additionally, the GeneAgent demonstrated notable advantages in generating highly similar names (Table 2). Specifically, GeneAgent generated 170 cases with a similarity greater than 90% and 614 cases exceeding 70%, compared to GPT-4’s 104 and 545 cases, respectively (Extended Data Fig. 1a). Remarkably, GeneAgent generated 15 names with a similarity score of 100%, while GPT-4 only generated three.

**Table 2:** Examples of gene sets that are assigned with different biological process names and similarity scores

Gene sets named by different methods.				
ID	Ground truth	GeneAgent	GPT-4	GSEA (performed via g:Profiler)
GO:0032459	Regulation of protein oligomerization	Protein sorting and lipid transport	Intracellular protein transport	Regulation of protein oligomerization
NeST:69	Protein nuclear transport	Nucleocytoplasmic Transport	Telomere maintenance and nuclear transport	Protein localization to nucleus
MSigDB:69	Peroxisome	Peroxisome protein	Peroxisome biogenesis	Protein localization to peroxisome

Gene sets named by GeneAgent with different similarity scores. Their direct ancestors in GO terms are obtained by g:Profiler.					
ID	Ground truth	GeneAgent	Similarity score	Direct ancestor in GO Terms	Similarity with ancestor (comparison with similarity score)
GO:0035108	Limb morphogenesis	Limb morphogenesis	1.000^a^	Limb development	0.928$\,{\boldsymbol{\downarrow }}$
GO:0015888	Thiamine transport	Thiamine transport and metabolism	0.989^a^	Vitamin transport	0.815$\,{\boldsymbol{\downarrow }}$
MSigDB:69	Peroxisome	Peroxisome protein	0.957^a^	Peroxisome organization	0.915$\,{\boldsymbol{\downarrow }}$
GO:0048319	Axial mesoderm morphogenesis	Mesodermal commitment pathway	0.772	Mesoderm morphogenesis	0.829$\,{\boldsymbol{\uparrow }}$^a^
NeST:61	Cullin–RING ubiquitin ligase complex	Ubiquitin- mediated proteolysis	0.826	Ubiquitin ligase complex	0.910$\,{\boldsymbol{\uparrow }}$^a^
NeST:8	Immune system	Lymphocyte activation	0.746	Leukocyte activation	0.929$\,{\boldsymbol{\uparrow }}$^a^
MSigDB:56	Reactive oxygen species pathway	Response to oxidative stress	0.721	Response to stress	0.911$\,{\boldsymbol{\uparrow }}$^a^

^a^The number indicates a proposed name is more similar to ground truth or more similar to the ancestors of ground truth.

Finally, we illustrated the practical significance of similarity scores by investigating the difference between generated names and their ground truths. As shown in Table 2, a similarity score exceeding 90% indicates the generated name has only minor differences, such as the addition of ‘Metabolism’. Similarity scores between 70% and 90%, however, typically indicate broader concepts, which would be more similar to an ancestor term of the ground truth. To confirm that this observation represents a true tendency, we conducted a hierarchical similarity analysis^8^ on gene sets within the GO dataset (Methods). Focusing on similarity scores ranging from 70% to 90%, we found that 75.4% of gene sets (303 of 402) had higher similarity scores with an ancestor term of the ground truth (Extended Data Fig. 1b).

### GeneAgent generates process names closer to ground truth

**Extended Data Fig. 2:**
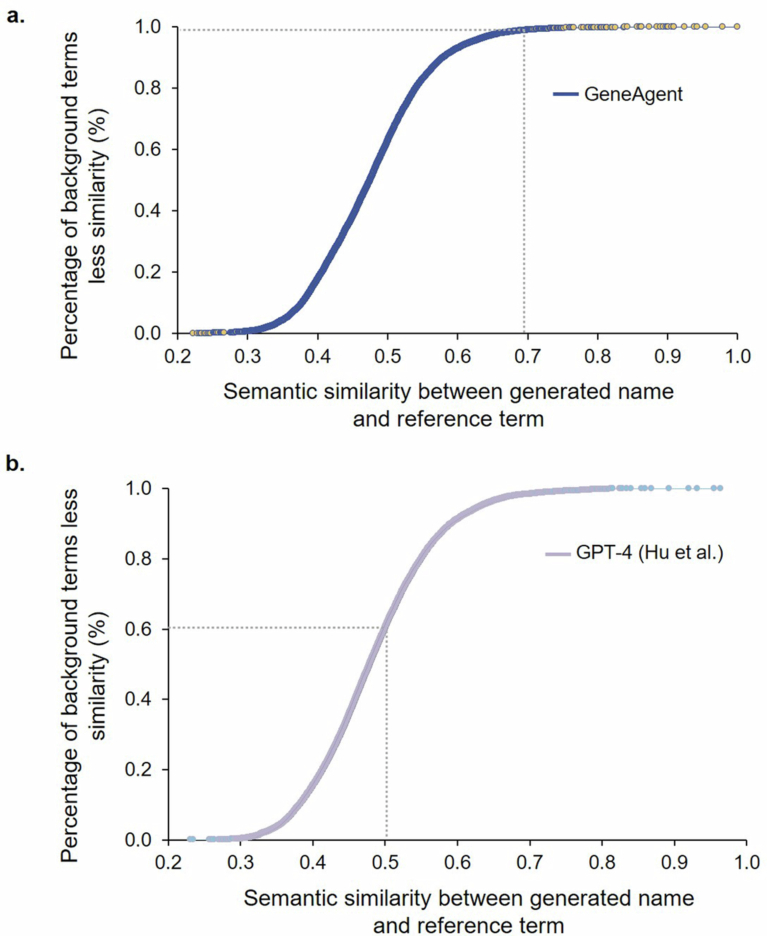
Semantic similarity between the generated name and ground truth is converted to the percentage of all terms in the background set with lower similarity to the generated name. **Semantic similarity between the generated name and ground truth (gray dashed line, x-axis) is converted to the percentage of all terms in the background set with lower similarity to the generated name (gray dashed line, y-axis). a**, Example of the ground truth (‘regulation of cardiac muscle hypertrophy in response to stress’) and the generated name of GeneAgent (‘Regulation of Cellular Response to Stress’). The similarity of ground truth and generated name is 0.695, which is higher than other 98.9% candidate terms in the background set. **b**, Example of the ground truth (‘regulation of cardiac muscle hypertrophy in response to stress’) and the generated name of GPT-4 (‘Calcium Signaling Pathway Regulation’). The similarity of ground truth and generated name is 0.500, which is higher than other 60.2% candidate terms in the background set.

Hu et al.^16^ introduced the ‘background semantic similarity distribution’ method, which evaluates the percentile ranking of the similarity score between the generated name and its ground truth within a background set of candidate terms. A high percentile indicates that the generated name is more semantically similar to the ground truth than the majority of candidate terms. To assess the performance of GeneAgent, we designed a similar pipeline based on MedCPT (Methods) and compared it to GPT-4. For example, GeneAgent generated the process name ‘regulation of cellular response to stress’ for the gene set with the ground-truth name ‘regulation of cardiac muscle hypertrophy in response to stress’, which achieved a similarity at the 98.9th percentile (Extended Data Fig. 2a), while GPT-4’s generated name, ‘calcium signaling pathway regulation’, ranked only at the 60.2nd percentile (Extended Data Fig. 2b).

Across the 1,106 gene sets tested, we identified cases where the similarity score between the generated name and the ground truth ranked within the top 90th percentile of 12,320 candidate terms (Fig. 2c). GeneAgent outperformed GPT-4 (Hu et al.^16^), with 76.9% (850) of the names generated by GeneAgent achieving semantic similarity scores in the 90th percentile. Specifically, GeneAgent produced 758 from GO, 46 from NeST and 46 from MSigDB. In contrast, GPT-4 (Hu et al.^16^) yields 742, 42 and 40 gene sets, respectively, from the same databases, amounting to 74.5% overall. GeneAgent’s advantage becomes more pronounced for high percentiles: at the 98th percentile, GeneAgent generated over 675 gene sets surpassing this threshold, compared to 598 for GPT-4. Notably, GeneAgent generated 82 gene sets that achieved a 100th percentile ranking, while GPT-4 achieved this for only 43 gene sets.

### GeneAgent generates an informative gene function summary

Building on the approach introduced in SPINDOCTOR^22^, which summarizes multiple biological processes based on gene function descriptions, we conducted enrichment testing on MSigDB. For this analysis, the verification reports produced during GeneAgent’s self-verification step served as the gene function synopsis (Methods). For comparison, we collected the narrative and ontological synopsis of 56 gene sets in MSigDB from the SPINDOCTOR study and evaluated results under the basic setting, where no gene synopsis was provided. For this test, we used GPT-4 with the summarization prompts provided by SPINDOCTOR (Supplementary Document 1).

To evaluate the accuracy of enrichment terms summarized from different gene synopses against those from conventional GSEA, we utilized g:Profiler^27^ to extract significant enrichment terms (*P* value ≤ 0.05) as ground truth for comparison. Then, we quantified the degree of overlap between the LLM-generated terms and significant terms (Methods). Using an exact match criterion, our findings reveal that 80.7% (296 of 367) of the LLM-generated terms aligned with significant enrichment terms when using verification reports as the gene synopsis (Fig. 2d). This proportion declines to 68.8% (282 of 410) when using ontological synopsis and diminishes to 56.0% (195 of 348) without using gene synopsis. As discussed in the SPINDOCTOR study, unmatched terms may represent instances where the model fabricates a biological function, that is, a hallucination. Therefore, the lower proportion (19.3%) of unmatched terms in GeneAgent underscores its efficacy in mitigating hallucinations.

### GeneAgent mitigates hallucinations by self-verification

While Hu et al.^16^ found LLMs to generate useful gene-set functions and explanatory analyses, verifying these contents required human inspection. However, GeneAgent incorporates the proposed self-verification module, acting as an agent and autonomously interacting with domain databases to obtain relevant knowledge to support or refute the raw LLM output. Consequently, verifying GeneAgent output is substantially performed automatically.

**Fig. 3:**
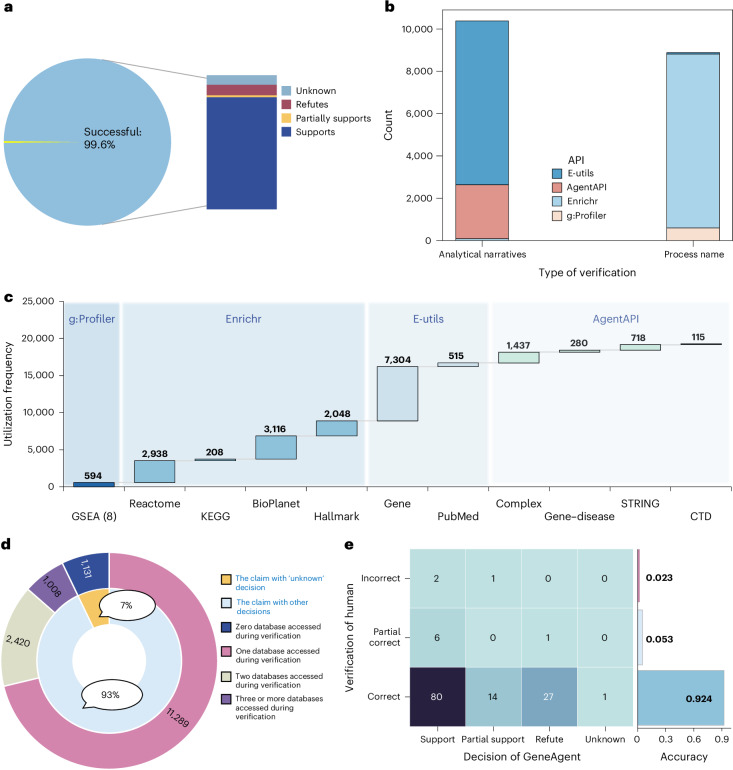
GeneAgent mitigates hallucinations by autonomously accessing Web APIs to interact with domain databases. **a**, Statistics of the outcome of the 15,903 claims collected from the 1,106 gene sets, including the proportion of the categorizations made by selfVeri-Agent. ‘Successfully’ denotes that the claims have a valid verification report returned by the selfVeri-Agent. **b**, Distribution (*y* axis) of four Web APIs in verifying Process name and Analytical narratives (*x* axis). **c**, The utilization frequency of different backend databases (*x* axis) in the self-verification stage of GeneAgent. **d**, Statistics of databases used to support the selfVeri-Agent to make decisions for the input claims. **e**, The results of human verification for the selected 132 claims derived from 10 gene sets. The inter-annotator agreement score was 93.9% (124/132), with a 95% Wilson score^40^ confidence interval ranging from 88.5% to 96.9%. Source data

To elucidate the role of self-verification, we examined 15,903 claims generated by GeneAgent and reported decisions of the selfVeri-Agent. Among these claims, 15,848 (99.6%) were successfully verified, with 84% supported, 1% partially supported, 8% refuted and the remaining 7% are unknown because the selfVeri-Agent output does not contain an explicit decision (Fig. 3a). A marginal fraction (0.4%) of claims were not verified due to the absence of gene names necessary for querying pertinent databases through Web APIs.

During the self-verification process, 16% of the claims were not supported. These unsupported claims were distributed across 794 gene sets, representing potential candidates for revision. Of these, 703 (88.5%) were subsequently modified.

Furthermore, we analyzed the frequency that the various Web APIs and their backend databases were utilized during the self-verification process. This analysis showed that process names are predominantly verified with Enrichr^28,29^ and g:Profiler APIs, whereas the validation of the explanatory analyses mainly relies on E-utils^30,31^ and AgentAPI (Fig. 3b). Additionally, GeneAgent interacts with backend databases 19,273 times to verify 15,848 claims (Fig. 3c). For these successfully verified claims, the frequency of databases used in the selfVeri-Agent (Fig. 3d) demonstrated that each decision is underpinned by evidence retrieved from at least one database. To estimate the accuracy of the self-verification process of GeneAgent, we manually reviewed ten randomly selected gene sets from NeST with a total of 132 claims, which received 88 supports, 15 partial supports, 28 refutes and one unknown by GeneAgent (Fig. 3e). Manual inspection demonstrated that 92% (122) of GeneAgent’s decisions are correct, indicating a high performance in self-verification (Supplementary Document 2).

### GeneAgent offers insightful explanations for novel gene sets

As a real-world utilization case, we applied GeneAgent to seven gene sets derived from the study of sub-clonal evolution on gene expression in mouse B2905 melanoma cell lines^32^ (Methods), with the number of genes in each set ranging from 19 to 49 (Table 1). These gene sets are identified from three subclones to the immunotherapy response, that is, high aggression and resistant (HA-R), high aggression and sensitive (HA-S) and low aggression and sensitive (LA-S). The results (Table 3) demonstrate that GeneAgent outperforms GPT-4 in generating correct process names and drafting informative explanation analysis.

**Table 3:** The comparison analysis of different methods in the case study

Results of GeneAgent and GPT-4 in the case study.			
ID	GPT-4	GeneAgent	Gene coverage
mmu05171 (HA-R)	Ribosomal protein synthesis	Cytosolic ribosome and protein synthesis	33/36
mmu03010 (HA-R)	Ribosomal protein synthesis and assembly	Cytosolic ribosome	34/35
mmu03010 (HA-S)	Ribosomal protein synthesis	Cytosolic ribosome	13/49
mmu05171 (HA-S)	Ribosomal protein synthesis	Cytosolic ribosome assembly and protein synthesis	47/47
mmu04015 (HA-S)	MAPK/ERK pathway regulation	Rap1 signaling pathway	27/27
mmu05100 (HA-S)	Caveolae-mediated endocytosis and actin remodeling	Bacterial invasion of epithelial cells	19/19
mmu05022 (LA-S)	Oxidative phosphorylation and neurodegeneration	Neurodegeneration and respiratory chain complex	23/24

Human annotation for the output of GeneAgent and GPT-4.										
ID	Better output annotated by genomic experts									
	Relevance		Readability		Consistency		Comprehensive		Final Decision	
	GPT	GAgent	GPT	GAgent	GPT	GAgent	GPT	GAgent	GPT	GAgent
mmu05171 (HA-R)		**✓**								**✓**
mmu03010 (HA-R)		**✓**								**✓**
mmu03010 (HA-S)									**×**	**×**
mmu05171 (HA-S)		**✓**				**✓**		**✓**		**✓**
mmu04015 (HA-S)		**✓**								**✓**
mmu05100 (HA-S)					**✓**		**✓**		**✓**	
mmu05022 (LA-S)		**✓**	**✓**					**✓**		**✓**

‘✓’ denotes the results judged to be better. ‘×’ denotes incoherent output. ‘Blank cell’ denotes both GPT-4 and GeneAgent perform well. ‘GPT’ and ‘GAgent’ indicate GPT-4 and GeneAgent, respectively.

**Extended Data Fig. 3:**
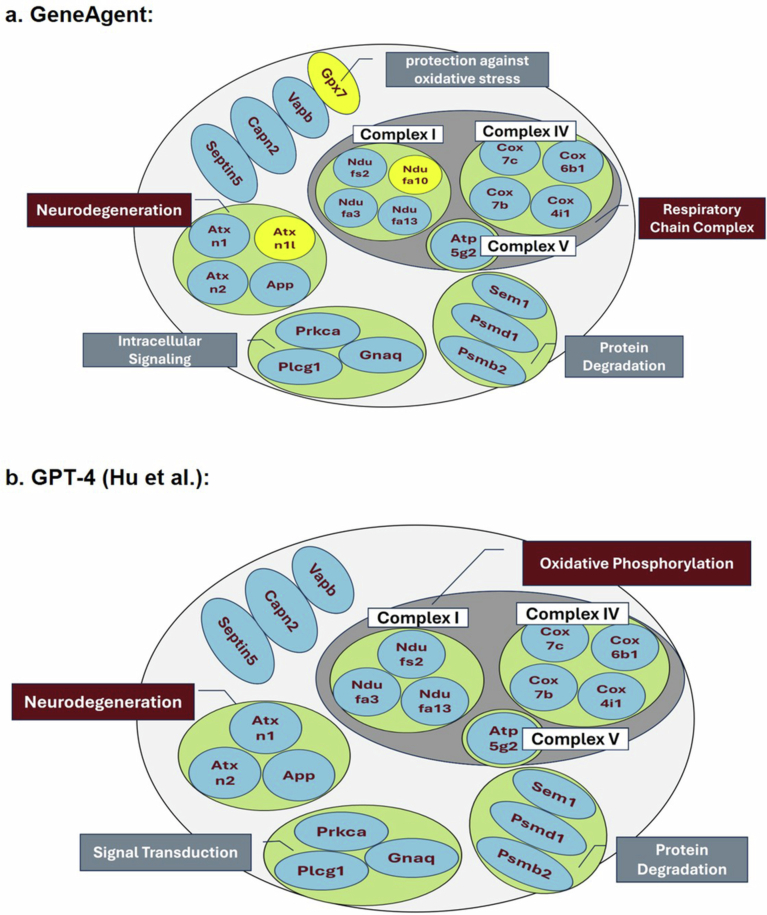
The example shown for the gene set named ‘mmu05022 (LA-S)’ in the case study. **a**, GeneAgent takes the ‘Neurodegeneration and Respiratory Chain Complex’ as the most prominent biological process. Complex I is the ubiquinone oxidoreductase, Complex IV is the cytochrome c oxidase and Complex V is the ATP synthase. **b**, GPT-4 (Hu et al.) takes the ‘Oxidative Phosphorylation and Neurodegeneration’ as the most prominent biological process.

Specifically, two gene sets, that is, mmu04015 (HA-S) and mmu05100 (HA-S), are assigned with process names that exhibit perfect alignment with the ground truth established by the domain experts (Table 3). Moreover, GeneAgent reveals novel biological insights for specific genes in the gene set. For example, for mmu05022 (LA-S), GeneAgent suggests gene functions related to subunits of complexes I, IV and V in the mitochondrial respiratory chain complexes^33^, and further summarizes the ‘respiratory chain complex’ for these genes (Extended Data Fig. 3a). However, GPT-4 categorizes these genes as ‘oxidative phosphorylation,’ which is a high-level biological process based on the mitochondrial respiratory chain complexes^34,35^, without including the gene *Ndufa10* (encoding NADH: ubiquinone oxidoreductase subunit A10) in this process. Similarly, GPT-4 does not include the gene *Atxn1l* (encoding ataxin 1-like) into ‘neurodegeneration’ and does not provide a biological function for the gene *Gpx7* (encoding glutathione peroxidase 7; Extended Data Fig. 3b). These results suggest that GeneAgent is more robust than GPT-4 for novel gene sets, and that GeneAgent is applicable to nonhuman genes.

To further measure the quality of outputs generated by GeneAgent and GPT-4, we formulated four criteria that are recognized as critical for practical use by genomic researchers: relevance, readability, consistency and comprehensiveness (Methods). We recruited two experts specializing in preclinical therapy testing, with a focus on genomic and immunological analysis, to manually assess and compare the results (Table 3b). GeneAgent and GPT-4 both demonstrate excellent readability and consistency across many cases. However, GeneAgent outperforms GPT-4 in relevance and comprehensiveness, which can be attributed to its ability to access domain-specific databases during the verification stage, thereby offering potentially valuable insights for experts. Nonetheless, there is one case, that is, mmu03010 (HA-S), where neither GeneAgent nor GPT-4 produces satisfactory results based on the four criteria. GeneAgent generates a narrow process name, ‘cytosolic ribosomes,’ that does not cover mitochondrial ribosomal genes such as *Mrpl10* (encoding mitochondrial ribosomal protein L10) and *Mrps21* (encoding mitochondrial ribosomal protein S21), while GPT-4 generates a hallucinated response, ‘synthesis’ (Supplementary Document 3).

## Discussion

### Self-verification in GeneAgent

**Extended Data Fig. 4:**
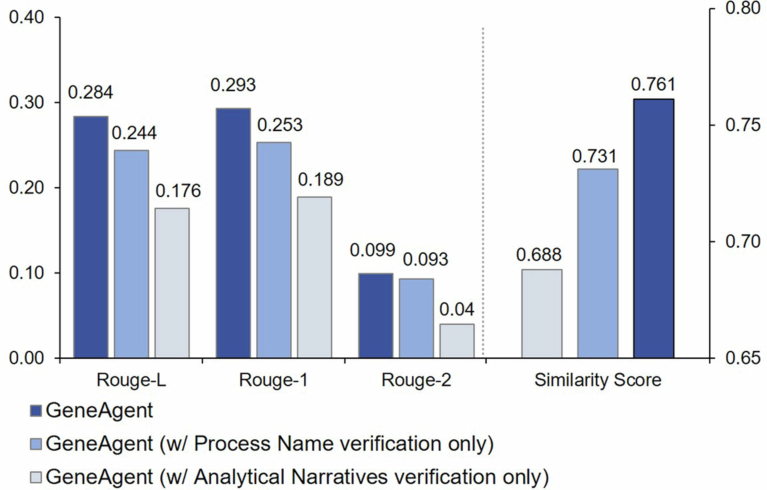
Ablation experiments for the Self-verification in GeneAgent on the NeST dataset. The plot shows the averaged ROUGE scores (left) and the averaged similarity scores (right) of GeneAgent with different settings among all gene sets in different datasets. GeneAgent (w/ Process Name verification only) denotes the GeneAgent is only configured with the verification for the process name of the raw output. GeneAgent (w/ Analytical Narratives verification only) denotes the GeneAgent is only configured with the verification for the analytical narratives of the raw output. GeneAgent denotes the method proposed in our study. Source data

Recent research has increasingly focused on the ‘self-verification’ within LLMs^19,36–38^. These studies utilize the same LLM to generate and then verify its own output, which may lead to overconfidence. This approach also has the potential to increase the risk of failing to discover novel insights, as the model might not adequately question or critique their initial findings^39^. In contrast, GeneAgent leverages established knowledge from manually curated domain-specific databases to verify the raw output of LLMs (Fig. 1b), which can not only mitigate the overconfidence in the initial results but also help reduce the potential for hallucination, thereby enhancing the reliability of LLMs. We performed an ablation experiment for the verification strategy used in the GeneAgent (Extended Data Fig. 4), with results that support the effectiveness of our self-verification setting.

### GeneAgent versus GSEA

**Extended Data Fig. 5:**
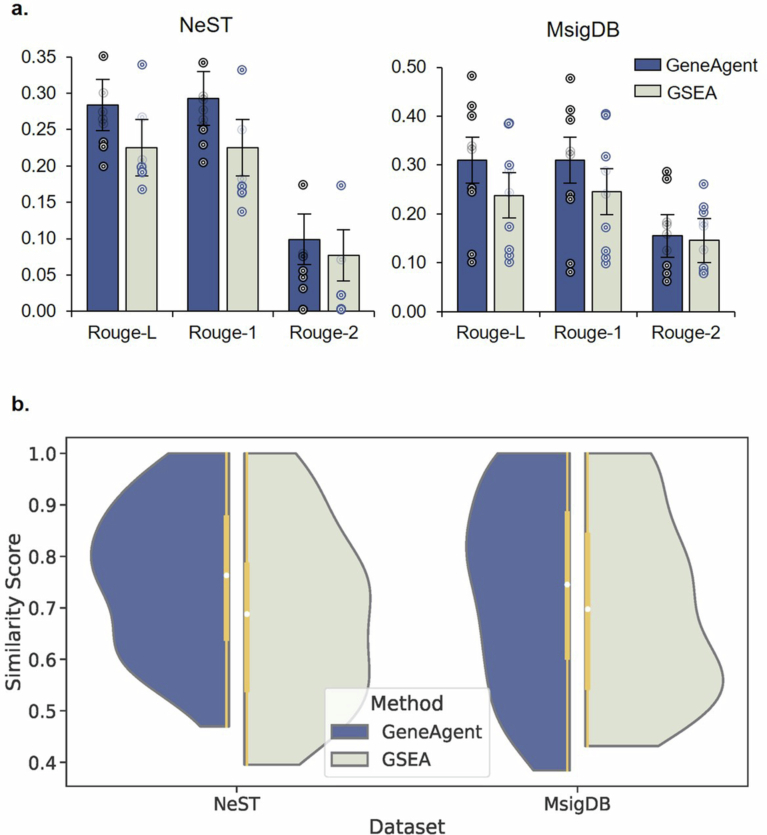
Comparison between GeneAgent and conventional GSEA method on the NeST (n = 50) and the MsigDB (n = 56) datasets. **a**, ROUGE scores obtained by GeneAgent and GSEA. The standard deviation (SD) on each bar was calculated using 9-fold cross-validation based on batch size (bs) sampling, with bs = 20 for both NeST and MSigDB. The ROUGE scores corresponding to each batch size are also presented in the figure. The central value of the error bars represents the mean score across all samples. The results are presented as mean ± SD. **b**, Distribution of similarity scores obtained by GeneAgent and GSEA. The middle points represent the mean values; bounds of the inner boxes of each violin plot represent the upper and lower percentiles; and whiskers represent the minimum and maximum points within all data samples. The statistic significant p-values are calculated by a one-tailed T test with 95% confidence intervals. The specific p-value for 106 gene sets is 4.5 × 10^−5^. The GSEA results are reproduced by the g:Profiler API. Source data

As an indispensable tool for gene-set analysis, GSEA produces significant enrichment terms and statistical information for gene sets, which can provide informative evidence to verify the raw output of an LLM. In GeneAgent, we included four different APIs (for example, g:Profiler) to ascertain the agreement of gene sets with those represented by expert-curated databases. By comparing the generated names and the most significant enrichment term produced by GSEA, we found that GeneAgent surpasses GSEA in terms of similarity and ROUGE scores (Extended Data Fig. 5). In addition to superior performance, GeneAgent can generate associated explanations, which increases the transparency of the results and explains the functions of genes in the proposed biological process. Therefore, GeneAgent can be seen as a system that merges the strengths of both LLMs and GSEA, delivering performance that surpasses each individual system.

### Importance of expert-curated domain databases

In addition to the eight databases from the GSEA tool, we have incorporated four databases for pathway analysis and six for gene functional verification (Fig. 3c). These databases formed a cohesive system that facilitates the discovery of gene-set knowledge by providing a reliable foundation of gene functions. The databases used in GSEA are complemented by the others, especially for examining the consistency of individual genes and their shared functions. This is particularly vital for uncovering latent biological functions among multiple genes, as it offers detailed insights into the characteristics of individual genes. Taken together, the domain-specific databases curated by experts are essential for enhancing the effectiveness of GeneAgent in discovering gene-set knowledge.

### Error analysis

We showed three representative cases in gene sets with a low similarity score across three datasets and their verification reports (Supplementary Table 3). The suboptimal performance of GeneAgent in those cases can be primarily attributed to two factors: (1) the erroneous rejection of an accurate process name due to the scale of domain databases is limited in self-verification; and (2) the incorrect endorsement of an originally dissimilar process because the selfVeri-Agent calls an unmatched database during verification. Using additional relevant domain databases in the self-verification stage or engineering a more effective prompt for the modification stage may help alleviate such issues.

### Limitations

In this work, we only selected GPT-4 as the backbone model, given its popularity and strong overall performance. While future work may explore other LLMs, Hu et al.^16^ show that GPT-4 outperforms GPT-3.5, Gemini-Pro, Mixtral-Instruct and Llama 2. Our work shows that while the self-verification step is effective, GeneAgent might still generate biological process names that are substantially different from their ground truth. Although ROUGE is a commonly used evaluation metric, it is not sufficient on its own to fully assess gene-set analysis tasks. Instead, it can serve as a supplement to the evaluation of semantic similarity. Nonetheless, GeneAgent demonstrates remarkable robustness across gene sets of different species and effectively mitigates hallucinations by automatically interacting with domain-specific databases.

## Methods

### Model setting and data collection

In this work, we utilized GPT-4 (version 20230613) as the backend model via the Azure OpenAI API, which is trained on data compiled before September 2021. The Azure OpenAI API is compliant with the Health Insurance Portability and Accountability Act, ensuring robust data privacy protection. To ensure stable and reproducible output, we set the temperature parameter to the absolute 0. The evaluated gene sets (Table 1) were derived from recent releases by Hu et al.^16^ and Joachimiak et al.^22^ after 2023. The gene set corresponding to each ground truth in different datasets was determined by aggregating the genes with which it was directly annotated with those of all its ontological descendants.

Furthermore, we collected 191 gene sets from PubMed articles that were published from November 2021 to December 2023 to evaluate the performance of different LLMs in various data sources (Supplementary Table 1). These gene sets range in size from 3 to 408, with an average of 35.67. The ground truths of these gene sets are released along with articles. The average word count in ground truth is 3.267. For model assessments, we developed the evaluation pipeline using Python (version 3.11.0) alongside PyTorch (version 1.13.0). Other necessary Python packages are NumPy (version 1.26.3), Pandas (version 2.1.4) and Seaborn (version v0.13.2).

### Overview of GeneAgent

GeneAgent is a language agent built upon GPT-4 to automatically interact with domain-specific databases to annotate functions for gene sets, which is composed of four key steps: generation, self-verification, modification and summarization. Each module is triggered by a specific instruction tailored to its function (Supplementary Document 1). The goal of GeneAgent is to generate a representative biological process name (*P*) for a set of genes, denoted as $D=\{{{g}_{i}|}_{i=1}^{N}\}$. Each gene *g*_*i*_ in this set is identified by its unique name, and the *D* is associated with a specific curated biological term, that is, ground truth (*G*). When provided with a *D*, GeneAgent outputs a $P$, accompanied by analytical texts (*A*) detailing the functions of the genes involved, which can be formally defined as GeneAgent (*D*) = (*P*, *A*).

### Pipeline of generating prominent biological process names

The gene set in the dataset *D* is separated by a comma (‘,’) and serves as input parameters for the instruction of the generation (*g*) step. Following the generation stage, *D* is assigned with an initial process name $({P}_{\rm{ini}})$ and corresponding analytical narratives ${(A}_{{ini}})$, that is, ${\rm{GeneAgent}}_{g}(D)=({P}_{\rm{ini}}\,,\,{A}_{\rm{ini}})$.

Afterwards, GeneAgent generates a list of claims for ${P}_{{ini}}$ by using statements like ‘be involved in’ and ‘related to’ to generate a hypothesis for the gene set and its process name. After that, GeneAgent activates selfVeri-Agent (Fig. 1c) to verify each claim in the list. Initially, selfVeri-Agent extracts all gene symbols and the process name from the claims. Subsequently, it utilizes the gene symbols to invoke the appropriate APIs for the autonomous interaction with domain-specific databases, using their established knowledge to validate the accuracy of the process name. Finally, it assembles a verification report (${{\mathcal{R}}}_{P}$) containing findings and decisions (that is, ‘supported’, ‘partially supported’ or ‘refuted’) to the input claim.

Next, GeneAgent initiates the modification (*m*) step to either revise or retain the ${P}_{{ini}}$ based on the findings in the ${{\mathcal{R}}}_{P}$. If the ${P}_{\rm{ini}}$ is determined to revise by GeneAgent, the ${A}_{\rm{ini}}$ is also instructed to be modified accordingly, that is, ${\rm{GeneAgent}}_{m}\left({P}_{\rm{ini}},{{A}}_{\rm{ini}},{{\mathcal{R}}}_{P}\right)=({P}_{\mathrm{mod}},\,{A}_{\mathrm{mod}})$. Following this, GeneAgent applies the self-verification to the ${A}_{\mathrm{mod}}$ to verify the gene functions in the explanatory analyses while checking the updated process name again. This step is also started with generating a list of claims for different gene names and their function names and is finished with deriving a new verification report (${{\mathcal{R}}}_{A}$) containing a decision of ‘supported’, ‘partially supported’ or ‘refuted’ made by the selfVeri-Agent.

Finally, based on the report ${{\mathcal{R}}}_{A}$, both ${P}_{\mathrm{mod}}$ and ${A}_{\mathrm{mod}}$ are modified according to the summarization (*s*) instruction to generate the final biological process name (*P*) and the analytical narratives (*A*) of gene functions, that is, ${\rm{GeneAgent}}_{s}\,({P}_{\mathrm{mod}},{A}_{\mathrm{mod}},\,{{\mathcal{R}}}_{A})=(P,A)$.

### Domain-specific databases configured in the selfVeri-Agent

In the self-verification stage, we configured four Web APIs to access 18 domain databases (Fig. 3c and Supplementary Document 4).

(1) g:Profiler^27^ (https://biit.cs.ut.ee/gprofiler/page/apis/) is an open-source tool for GSEA. In GeneAgent, we used eight domain-specific databases: GO, KEGG^41^, Reactome^42^, WikiPathways^43^, Transfac^44^, miRTarBase^45^, CORUM^46^ and Human Phenotype Ontology^47^ to perform enrichment analysis for the gene set. For each gene set, we used the g:GOSt interface to identify the top five enrichment terms along with their descriptions.

(2) Enrichr^28,29^ (https://maayanlab.cloud/Enrichr/help#api/) is also a valuable tool for GSEA. We configured four databases related to the pathway analysis in the Enrichr API, that is, KEGG_2021_Human, Reactome_2022, BioPlanet_2019 (ref. ^48^) and MSigDB_Hallmark_2020. In GeneAgent, we selected to return the top five standard pathway names via databases.

(3) E-utils^30,31^ (https://www.ncbi.nlm.nih.gov/) is an API designed for accessing the NCBI databases for various biological data. In GeneAgent, we augment our repository of functional information associated with an individual gene by invoking its Gene database and PubMed database. Different databases can be used by defining the database parameter as Gene or PubMed in the foundation API.

(4) AgentAPI is our custom API library, developed using four gene-centric databases related to gene–disease, gene–domain, PPI and gene–complex. GeneAgent calls the appropriate database by specifying the desired interface at the end of the basic API, and subsequently retrieving the top ten relevant IDs to gene functions. These IDs are then used to match their names in the corresponding database.

Notably, we implemented a masking strategy for APIs and databases during the self-verification stage to ensure unbiased assessments across various gene sets. Specifically, we removed the g:Profiler API when assessing gene sets collected from the GO dataset because it can perfectly derive their ground truths by accessing the GO database. Similarly, we masked the ‘MSigDB_Hallmark_2020’ database within the Enrichr API when evaluating gene sets collected from the MSigDB database.

### Calculation of ROUGE score

Three distinct ROUGE metrics^25^ are used to access the recall of generated names relative to ground truths: that is, ROUGE-1 and ROUGE-2, which are based on n-gram, and ROUGE-L, which utilizes the longest common subsequence (LCS). The calculation formulas are as follows:

$${\rm{ROUGE}}-{\rm{N}}=\frac{{\sum }_{S\in {\rm{ref}}}{\sum }_{{g}_{N}\in S}{\rm{count}}_{\rm{match}}\left({g}_{N}\right)}{{\sum }_{S\in {\rm{ref}}}{\sum }_{{g}_{N}\in S}{\rm{count}}\left({g}_{N}\right)},\text{N}=1,\,2$$

$$\left\{\begin{array}{c}{R}_{\rm{lcs}}=\frac{\rm{LCS}\left({\rm{ref}},{\rm{hyp}}\right)}{m}\\ {P}_{\rm{lcs}}=\frac{{\rm{LCS}}\left({\rm{ref}},{\rm{hyp}}\right)}{n}\\ {\text{ROUGE}}-{\text{L}}\,=\frac{\left(1+{\beta }^{2}\right){R}_{\rm{lcs}}{P}_{\rm{lcs}}}{{R}_{\rm{lcs}}+{\beta }^{2}{P}_{\rm{lcs}}}\end{array}\right.$$

Here, the ‘ref’ denotes the reference terms and ‘hyp’ denotes the generated names. *m* and *n* are the token lengths of ‘ref’ and ‘hyp’, respectively. *β* is a hyper-parameter.

### Calculation of semantic similarity

After generating the biological process name (*P*) for the gene set *D*, the semantic similarity between $P$ and its ground truth (*G*) is computed by MedCPT^26^, a state-of-the-art model for language representation in the biomedical domain. It is built based on PubMedBERT^49^ with further training using 255 million query–article pairs from PubMed search logs. Compared with SapBERT^50^ and BioBERT^51^, MedCPT has higher performance in encoding the semantics of biomedical texts.

- Calculation of semantic similarity between *P* and *G*
  First, *P* and *G* are encoded by MedCPT into embeddings, and then the cosine similarity between their embeddings is calculated, yielding a score in the interval [−1, 1]. Finally, we take the average value of all similarity scores to evaluate the performance of GeneAgent on gene sets in each dataset.
- Calculation of background semantic similarity distribution

First, *P* is paired with all possible terms ${G}_{i}{\mathcal{\in}}{\mathcal{Q}}$, where *Q* denotes 12,320 candidate terms consisting of 12,214 GO biological process terms, and all available terms in NeST (50) and MSigDB (56). Then, *P* and *G*_*i*_ are fed into MedCPT to get the embeddings, that is, $\mathop{P}\limits^{ \rightharpoonup }$ and $\mathop{{G}_{i}}\limits^{ \rightharpoonup }$. Afterwards, we calculated the cosine similarity for each $< \mathop{P}\limits^{ \rightharpoonup },\mathop{{G}_{i}}\limits^{ \rightharpoonup } >$ pair. Finally, we ranked all cosine scores from large to small and observed the position where the pair <*P*,*G*_*p*_> (*G*_*p*_ is the ground truth for *P*) located in. The higher position denotes the generated names have a higher similarity score to their ground truths than other candidate terms.

### Calculation of hierarchical semantic similarity

We first collected the hierarchical structures of all GO terms from the GoBasic.obo file in the GO database (2023-11-15 version), yielding 1,951,615 GO term pairs across five relationships: ‘is a’, ‘part of’, ‘regulates’, ‘negatively regulates’ and ‘positively regulates’. Next, we extracted the ancestral terms for all ground truths in the evaluation datasets used in this study, limiting the distance from each ground truth to its respective ancestor to within three hops. Finally, we calculated the semantic similarity between each ancestral term and its corresponding ground truth to assess whether the generated names achieved a higher similarity score with ancestral terms. For our evaluation, we computed hierarchical semantic similarity exclusively for gene sets in the GO dataset.

### Pipeline of enrichment term test using verification reports

For gene sets in MSigDB, we first collected its verification report produced by the selfVeri-Agent of GeneAgent. Afterwards, each gene set and the associated report were used as the parameters of the instruction (Supplementary Document 1) for the GPT-4. Therefore, GPT-4 can summarize multiple enrichment terms for the given gene set. Finally, we used the exact match to evaluate the accuracy of the tested terms summarized by the GPT-4. Specifically, for each gene set in the evaluation, we first utilized g:Profiler to perform GSEA, where the *P*-value threshold is set to 0.05. Then, we obtained significant enrichment terms for the given gene sets as the ground truth. Finally, we counted the number of tested terms summarized by GPT-4 that correctly match the significant enrichment term of each gene set. One tested term is considered as accurate only when there is an exact match between all the words in the tested term and one term in the ground truth.

### Human checking for the decisions of selfVeri-Agent

We randomly selected ten gene sets from NeST with 132 claims for human inspection. There are two parts in the verification report: the claims and the decisions to the claims along with evidence (Supplementary Document 2). Annotators were asked to label the selfVeri-Agent decisions (that is, support, partially support and refute) for each claim and judge whether such decisions are correct, partially correct or incorrect, which follows the study of natural language inference^52^ and fact verification^53^. For each claim, the annotators need to make a judgment based on assertions of the gene (set) functions provided in the evidence:

- **Correct**: This category applies when GeneAgent’s decision completely aligns with the evidence supporting the input claim. The decision is considered correct if it accurately reflects the evidence documented, demonstrating a clear and direct connection between the claim and the supporting data.
- **Partially correct**: It is designated when GeneAgent’s decision requires indirect reasoning or when the decision, although related, does not completely align with the direct evidence provided. This occurs when the decision is somewhat supported by the evidence but requires additional inference or context to be fully understood as supporting the input claim.
- **Incorrect**: This category is used when GeneAgent’s decision either contradicts the evidence or lacks any substantiation from the verification report.

### Melanoma gene sets in the preclinical study

The mouse B2905 melanoma cell line, which is derived from a tumor from the M4 model, where melanoma is induced by ultraviolet irradiation on pups of hepatocyte growth factor-transgenic C57BL/6 mice^54^.

Specifically, 24 single cells were isolated from the parental B2905 melanoma line and then expanded to become individual clonal sublines (that is, C1 to C24)^55^. Each of these 24 sublines was subjected to whole-exome sequencing and full-transcript single-cell RNA sequencing using the Smart-seq2 protocol. The single nucleotide variants called from exome sequencing results were used to build the tumor progression tree for all the 24 sublines. Based on the in vivo growth and therapeutic responses of the sublines in the clusters, three clades are named as ‘high aggressiveness and resistant (HA-R)’, ‘high aggressiveness and sensitive (HA-S)’ and ‘low aggressiveness and sensitive (LA-S)’^32^. Afterwards, EvoGeneX^56^ is applied to the single-cell RNA-sequencing data of the 24 clonal sublines, where the phylogenetic relation is defined by the mutation-based tumor progression tree, to identify adaptively upregulated and downregulated genes in each of HA-R, HA-S and LA-S clades. The adaptively upregulated and downregulated gene lists were then subjected to the Kyoto Encyclopedia of Genes and Genomes pathway enrichment analysis. The genes in the enrichments and their enriched terms are used to test the GeneAgent. In our case study, we only utilized the seven gene sets analyzed from the clonal subline as the evaluation data of GeneAgent. We did not access or process any original data from clinical experiments.

### Human annotation for outputs in the case study

For the assessment of different outputs between GeneAgent and GPT-4, we established four criteria following the existing studies on the evaluation of LLMs^57,58^.

- **Relevance**: Assess whether the content about genes pertinently reflects their functions, providing value to biologists.
- **Readability**: Evaluate the fluency and clarity of the writing, ensuring it is easily understandable.
- **Consistency**: Determine whether the analytical narratives align consistently with the specified process name.
- **Comprehensiveness**: Verify whether the outputs provide a comprehensive understanding of gene functions.

Based on these four established criteria, two experts are tasked with evaluating the final responses from the outputs of GPT-4 and GeneAgent. They operate the annotation under a blind assessment protocol, where they are unaware of the algorithm that produced each response. Their main responsibility is to annotate and compare the preference for outputs generated by GPT-4 versus GeneAgent. They carefully review and select the more effective response, justifying their selections with relevant comments. Following a comprehensive synthesis of all feedback, these two experts are required to make a definitive judgment on which output most effectively satisfies the users’ requirement (Supplementary Document 3).

### Reporting summary

Further information on research design is available in the Nature Portfolio Reporting Summary linked to this article.

## Online content

Any methods, additional references, Nature Portfolio reporting summaries, source data, extended data, supplementary information, acknowledgements, peer review information; details of author contributions and competing interests; and statements of data and code availability are available at 10.1038/s41592-025-02748-6.

## Supplementary information

Supplementary Information Supplementary Fig. 1 and Supplementary Tables 1–4

Reporting Summary

Supplementary Documents 1–5 Supplementary Document 1: The details of prompts used in GeneAgent and the related experiments. Supplementary Document 2: The details of claims and human checking for the self-verification stage of GeneAgent. Supplementary Document 3: The details of expert annotations for the case study. Supplementary Document 4: The details of access links and prompts used in API calling of GeneAgent. Supplementary Document 5: The demonstration of process names generated by GSEA and different LLMs.

Supplementary Code 1 The source code for GeneAgent.

Supplementary Data 1 The source evaluated gene sets.

## Source data

Source Data Fig. 2 The statistical experimental data for Fig. 2.

Source Data Fig. 3 The statistical experimental data for Fig. 3.

Source Data Extended Data Fig. 1 The statistical experimental data for Extended Data Fig. 1.

Source Data Extended Data Fig. 4 The statistical experimental data for Extended Data Fig. 4.

Source Data Extended Data Fig. 5 The statistical experimental data for Extended Data Fig. 5.

## Data Availability

Publicly available gene sets were used in this study. GO (2023-11-15 release) and the original selected NeST gene sets used in the study of Hu et al.^16^ are available at https://github.com/idekerlab/llm_evaluation_for_gene_set_interpretation/blob/main/data/. Gene sets used in the MSigDB dataset are the subset of data used in the research at https://github.com/monarch-initiative/talisman-paper/tree/main/genesets/human/. Processed gene sets evaluated in this study are provided at https://github.com/ncbi-nlp/GeneAgent/ and can be downloaded from the Zenodo repository^59^. Source data are provided with this paper.

## Code availability

The MedCPT (version 2023-10-25) used for the calculation of similarity scores is publicly available at https://github.com/ncbi/MedCPT/ and https://huggingface.co/ncbi/MedCPT-Query-Encoder/. The GeneAgent code has been released at https://github.com/ncbi-nlp/GeneAgent/ and can be accessed via Zenodo^59^. A demonstration website with open-access permissions is available at https://www.ncbi.nlm.nih.gov/CBBresearch/Lu/Demo/GeneAgent/.
