# Supplementary material for: GeneAgent: self-verification language agent for gene-set analysis using domain databases
Source: Nat Methods. 2025 Jul 28;22(8):1677–85. doi: 10.1038/s41592-025-02748-6 (PMC12328209; doi:10.1038/s41592-025-02748-6)
Supplement: Supplementary file 1 — Supplementary Fig. 1 and Supplementary Tables 1–4 [file 41592_2025_2748_MOESM1_ESM.pdf]

nature methods

Supplementary information

https://doi.org/10.1038/s41592-025-02748-6

GeneAgent: self-verification language agent for gene-set analysis using domain databases

In the format provided by the authors and unedited

Complementary Experiments

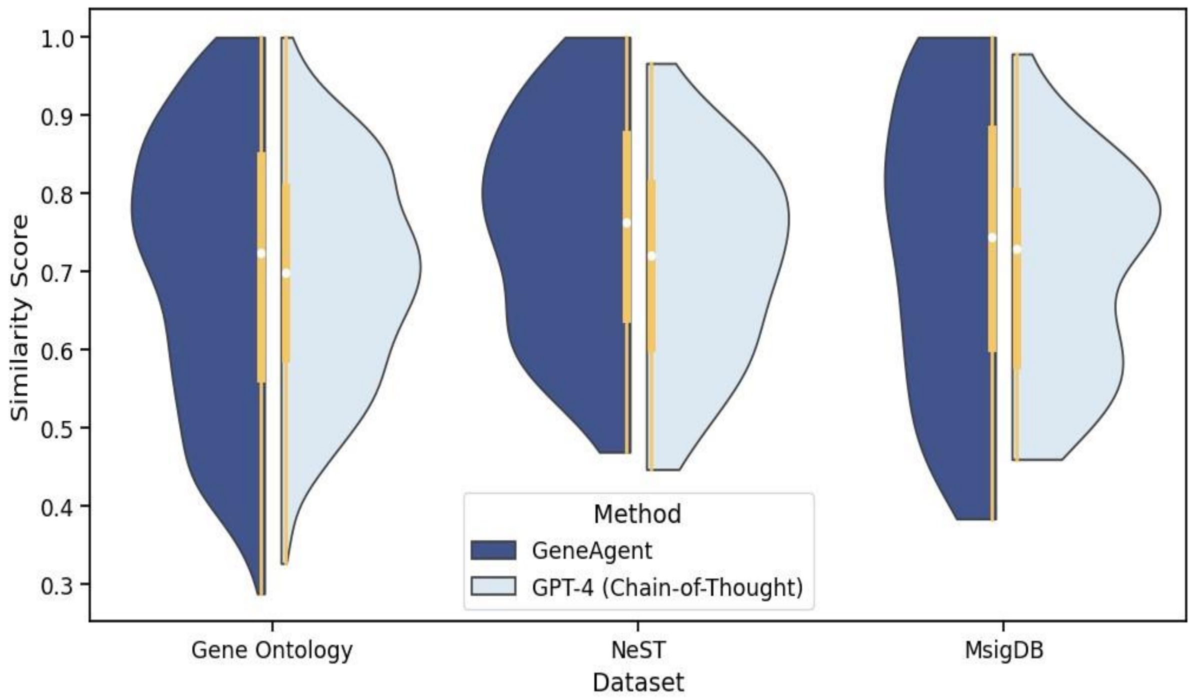

Supp. Fig. 1. GeneAgent versus GPT-4 with Chain-of-Thought (CoT). To improve the performance of GPT-4 on specific tasks, the CoT method emerges as a potent strategy in prompt engineering, which would deconstruct the task into multiple sequential steps informed by researchers' expertise. In pursuit of this, we crafted a CoT instruction for the standard GPT-4 and compared it to GeneAgent on three datasets. The results show that the similarity score of GPT-4 (CoT) is indeed slightly better than GPT-4, yet it remains inferior to GeneAgent. The total number of gene sets used for the statistic is 1,000 (Gene Ontology), 50 (NeST), and 56 (MsigDB). The middle points represent the mean values; bounds of the inner boxes of each violin plot represent the upper and lower percentiles; and whiskers represent the minimum and maximum points within all data samples. The statistic significant p-values are calculated by a one-tailed T test with 95% confidence intervals. The specific p-value for 1,106 gene sets is 3.4×10^{-3}.

Supp. Tab. 1. The performance comparisons among different LLMs. The best results are bolded. "*" denote the significant improvement compared with the second-best model. All results of LLMs (except for GeneAgent) are reproduced by only using the prompts proposed by Hu *et al*.

Model		Llama-3-8B	GPT-4	GPT-4o	GeneAgent (GPT-4)	GeneAgent (GPT-4o)
**Gene Ontology**	ROUGE-L	0.133	0.184	0.184	0.250	**0.260**
	ROUGE -1	0.146	0.201	0.207	0.262	**0.269**
	ROUGE -2	0.025	0.049	0.036	0.120	**0.126**
	Similarity Score	0.623	0.689	0.703	0.705	**0.720***
**NeST**	ROUGE -L	0.197	0.239	0.185	**0.284**	0.248
	ROUGE -1	0.210	0.252	0.200	**0.293**	0.265
	ROUGE -2	0.073	0.082	0.065	0.099	**0.121**
	Similarity Score	0.676	0.708	0.687	**0.761***	0.731
**MsigDB**	ROUGE -L	0.177	0.239	0.220	**0.310**	0.282
	ROUGE -1	0.177	0.239	0.220	**0.310**	0.289
	ROUGE -2	0.037	0.074	0.046	**0.155**	0.104
	Similarity Score	0.663	0.722	0.715	**0.736***	0.722
**PMDGenes**	ROUGE -L	0.116	0.168	0.163	0.367	**0.406**
	ROUGE -1	0.123	0.172	0.169	0.368	**0.407**
	ROUGE -2	0.019	0.073	0.063	0.269	**0.307**
	Similarity Score	0.610	0.637	0.635	0.727	**0.751***

Supp. Tab. 2 (Related to the Figure 1). A complete example for the workflow of GeneAgent. RED FONT denote the corrected contents after self-verification. GREEN FONT denote the retained contents after self-verification. BULE FONT denote the newly added contents while ~~**deleted lines**~~ denote the deleted contents.

Input gene set: ERBB2, ERBB4, FGFR2, FGFR4, HRAS, KRAS

1. Generation of raw output using standard LLMs

Process Name: Receptor Tyrosine Kinase Signaling

Analytical Narratives: The system of interacting proteins, encoded by the genes ERBB2, ERBB4, FGFR2, FGFR4, HRAS, and KRAS, primarily performs the biological process of receptor tyrosine kinase signaling.

1. ERBB2 and ERBB4: These genes encode for the proteins ErbB2 and ErbB4, respectively, which are members of the epidermal growth factor receptor (EGFR) family. **[...]**

2. FGFR2 and FGFR4: These genes encode for fibroblast growth factor receptors 2 and 4. **[...]**

3. HRAS and KRAS: These genes encode for the proteins H-Ras and K-Ras, respectively. **[...]**

In conclusion, the primary biological process performed by this system of interacting proteins is receptor tyrosine kinase signaling, **[...]**

2. self-Verification for the Process Name

Claims for process name: ERBB2, ERBB4, FGFR2, FGFR4, HRAS, KRAS is involved in RTK Signaling; ERBB2, ERBB4, FGFR2, FGFR4, HRAS, KRAS is associated with RTK Signaling; **[...]**

selfVeri-Agent:

Input Claim: ERBB2, ERBB4, FGFR2, FGFR4, HRAS, KRAS is involved in RTK Signaling

Verification Report: The claim is not directly verified (**i.e., refuted**) by selfVeri-Agent. The top enrichment function names of the given gene set include "MAPK signaling pathway," **[...]** while these functions are merely related to the name of "RTK Signaling". Therefore, based on the provided data, the claim cannot be confirmed.

[...]

3. Modification for process names in the Analytical Narratives

Process Name: MAPK signaling pathway

Analytical Narratives: The system of interacting proteins, encoded by the genes ERBB2, ERBB4, FGFR2, FGFR4, HRAS, and KRAS, primarily performs the biological process of MAPK signaling pathway.

1. ERBB2 and ERBB4: These genes encode for the proteins ErbB2 and ErbB4, respectively, which are members of the epidermal growth factor receptor (EGFR) family. **[...]**

2. FGFR2 and FGFR4: These genes encode for fibroblast growth factor receptors 2 and 4. **[...]**

3. HRAS and KRAS: These genes encode for the proteins H-Ras and K-Ras, respectively. **[...]**

In conclusion, the primary biological process performed by this system of interacting proteins is MAPK signaling pathway, **[...]**

4. self-Verification for Analytical Narratives

Claims for analytical narratives: ERBB2 and ERBB4 are members of the epidermal growth factor receptor (EGFR) family. FGFR2 and FGFR4 are part of the fibroblast growth factor receptor (FGFR) family. **[...]**

selfVeri-Agent:

Input Claim: ERBB2 and ERBB4 are members of the epidermal growth factor receptor (EGFR) family.

Verification Report: The claim is verified and is correct (**i.e., support**). Both ERBB2 and ERBB4 are indeed members of the epidermal growth factor receptor (EGFR) family of receptor tyrosine kinases (RTKs). Evidence: 1. ERBB2: The gene summary for ERBB2 states that it encodes a member of the epidermal growth factor (EGF) receptor family of receptor tyrosine kinases. This information is provided by (RefSeq, Jul 2008). 2. ERBB4: The gene summary for ERBB4 states that it is a member of the Tyr protein kinase family and the epidermal growth factor receptor subfamily. This information is provided by (RefSeq, Jul 2008).

5. Summarization for final Process Name and Analytical Narratives

Process Name: MAPK Signaling Pathway

Analytical Narratives: The proteins encoded by the genes ERBB2, ERBB4, FGFR2, FGFR4, HRAS, and KRAS are all integral components of the MAPK signaling pathway, crucial for cell growth, differentiation, and survival.

ERBB2 and ERBB4 are members of the epidermal growth factor receptor (EGFR) family. **[...]**

FGFR2 and FGFR4 are part of the fibroblast growth factor receptor (FGFR) family. **[...]**

HRAS and KRAS ~~encode for the proteins H-Ras and K-Ras, respectively~~ are GTPases that act as molecular switches in RTK signaling. **[...]**

In conclusion, the primary biological process performed by this system of interacting proteins is MAPK signaling pathway, **[...]**

1

Supp. Tab. 3. Gene sets analysis with low semantic similarity score. XXX (**) denotes the name generated by GPT-4 (Hu *et al*.) and the semantic similarity to the ground truth. <u>XXX</u> denotes the enrichment results returned by the domain databases.

ID	Ground Truth	Name generated by GeneAgent	Similarity Score	Major Evidence in the Verification Report	Basic Reason
NEST: 169	Neg Regulation EGFR	Cell Proliferation and Adhesion Regulation	0.470	The claim that the **EGFR Signaling Pathway Regulation (0.739)** is not directly supported by the data. The top 5 enrichment function names include <u>Endometrial cancer, Prostate cancer, Embryonic stem cell pluripotency pathways, and Breast cancer</u>.	Erroneous rejection of an accurate process name: The original similar process name generated by standard GPT-4 is incorrectly refuted during the verification for Process Name.
MsigDB: 12	Androgen Response	Cytoplasmic Protein Interaction and Regulation	0.384	[1] The gene set provided is indeed associated with **prostate cancer progression (0.615)**. The top 5 enrichment function names include <u>cytoplasm, prostate; glandular cells [High], prostate; glandular cells [≥Medium], extracellular exosome, and extracellular vesicle</u>.<br>[2] CDK6 is associated with the progression of prostate cancer cannot be verified.<br>[3] KLK2 and KLK3 genes are well-known biomarkers for prostate cancer cannot be fully verified.	Erroneous rejection of an accurate process name: The original similar process name is supported but it is refuted during the verification for Analytical Narratives.
GO: 0046684	response to pyrethroid	Catecholamine Biosynthesis	0.369	The claim that the process of **Catecholamine Biosynthesis (0.369)** is supported. The gene set DDC, TH is involved in several biological pathways related to <u>neurotransmitter disorders, dopamine metabolism, biogenic amine biosynthesis, and amine-derived hormones</u>. However, the gene SCN2B does not appear to be involved in these pathways.	Incorrect endorsement of an originally dissimilar process name: The original dissimilar process name generated by standard GPT-4 is incorrectly supported.

Supp. Tab. 4. Cost and time estimation of GeneAgent equipped with different GPT models.

Models	Cost per 1M tokens	Average tokens (Input/Output)	Estimated Cost per Gene Set	Estimated Average Running Time per Gene Set
GeneAgent (*GPT-4o*)	Input: $2.50<br>Cached Input: $1.25<br>Output: $10	~9000/~3600	$0.053	~5 min
GeneAgent (*GPT-4-32k*)	Input: $60<br>Output: $120	~6000/~2400	$0.648	~4 min
GeneAgent (*GPT-3.5-Turbo*)	Input: $1.50<br>Output: $2	~7500/~3650	$0.019	~5min
