# Supplementary material for: GeneAgent: self-verification language agent for gene-set analysis using domain databases
Source: Nat Methods. 2025 Jul 28;22(8):1677–85. doi: 10.1038/s41592-025-02748-6 (PMC12328209; doi:10.1038/s41592-025-02748-6)
Supplement: Supplementary file 2 — Reporting Summary [file 41592_2025_2748_MOESM2_ESM.pdf]

nature portfolio

Corresponding author(s): Zhiyong Lu, PhD

Last updated by author(s): Mar 6, 2025

Reporting Summary

Nature Portfolio wishes to improve the reproducibility of the work that we publish. This form provides structure for consistency and transparency in reporting. For further information on Nature Portfolio policies, see our Editorial Policies and the Editorial Policy Checklist.

Statistics

For all statistical analyses, confirm that the following items are present in the figure legend, table legend, main text, or Methods section.

n/a	Confirmed	
☐	☒	The exact sample size (*n*) for each experimental group/condition, given as a discrete number and unit of measurement
☐	☒	A statement on whether measurements were taken from distinct samples or whether the same sample was measured repeatedly
☐	☒	The statistical test(s) used AND whether they are one- or two-sided<br>*Only common tests should be described solely by name; describe more complex techniques in the Methods section.*
☐	☒	A description of all covariates tested
☒	☐	A description of any assumptions or corrections, such as tests of normality and adjustment for multiple comparisons
☐	☒	A full description of the statistical parameters including central tendency (e.g. means) or other basic estimates (e.g. regression coefficient) AND variation (e.g. standard deviation) or associated estimates of uncertainty (e.g. confidence intervals)
☐	☒	For null hypothesis testing, the test statistic (e.g. *F*, *t*, *r*) with confidence intervals, effect sizes, degrees of freedom and *P* value noted<br>*Give P values as exact values whenever suitable.*
☒	☐	For Bayesian analysis, information on the choice of priors and Markov chain Monte Carlo settings
☐	☒	For hierarchical and complex designs, identification of the appropriate level for tests and full reporting of outcomes
☒	☐	Estimates of effect sizes (e.g. Cohen's *d*, Pearson's *r*), indicating how they were calculated

Our web collection on statistics for biologists contains articles on many of the points above.

Software and code

Policy information about availability of computer code

Data collection	No software and code for the gene sets collection in Gene Ontology and NeST. These gene sets are directly collected from the Hu et al.'s study. We develop a preprocess code for changing the original gene sets separated by newlines in the MSigDB datasets as the gene sets delimited with the comma using the Python with 3.11.0 version.
Data analysis	In the GeneAgent, we utilized GPT-4 (version 20230613) as the backend model via the Azure OpenAI API, which is trained on data compiled before September 2021. Also, we employed several APIs to access the domain databases. First, we leveraged the g:Profiler (version e112_eg59_p19_25aa4782) to access the functional enrichment analysis databases. Second, we leveraged the Enrichr (version 20230608) to acess the pathway enrichment analysis databases. Finally, we leverage the E-utils (version 20181024) to access the gene summaries databases such as NCBI gene databases and PubMed articles.<br>We formulated several python scripts to evaluate the Rouge score, semantic similarity and exact match proportion. In these scripts, the python version is 3.11.0 along with the Pytorch of version 1.13.0. Other necessary python packages are Numpy (the version is 1.26.3) and Pandas (the version is 2.1.4). We also used the biomedicine text encoder MedCPT with the version of 20231025 (https://github.com/ncbi/MedCPT) to encode the text obtained by our developed methods for the semantic similarity evaluation. An available GitHub Gist containing all codes of data analysis is attached to the manuscript. For the demonstration of results, we also use the seaborn python package with the version of v0.13.2.<br>The complete source code to compile GeneAgent is available at https://github.com/ncbi-nlp/GeneAgent, and also can be downloaded by the Zenodo repository at DOI: 10.5281/zenodo.14976596.

For manuscripts utilizing custom algorithms or software that are central to the research but not yet described in published literature, software must be made available to editors and reviewers. We strongly encourage code deposition in a community repository (e.g. GitHub). See the Nature Portfolio guidelines for submitting code & software for further information.

Data

Policy information about availability of data

All manuscripts must include a data availability statement. This statement should provide the following information, where applicable:

- Accession codes, unique identifiers, or web links for publicly available datasets
- A description of any restrictions on data availability
- For clinical datasets or third party data, please ensure that the statement adheres to our policy

We conduct comparative experiments on gene sets from three distinct sources: literature curation (Gene Ontology), proteomics analysis (NeST system of human cancer proteins), and molecular functions (MSigDB). Specifically, to evaluate the recovery of literature-curated gene set functions, we used the same gene sets as those studied in the Hu's research (https://github.com/idekerlab/llm_evaluation_for_gene_set_interpretation/blob/main/data/), which contains 1000 terms randomly sampled from the GO Biological Process branch (GO-BP 2023-11-15 release). To evaluate the exploration of 'omics gene sets, we collected 50 gene sets from the Hu's research, where the genes encoding complexes of interacting proteins are identified by proteomic methods. Furthermore, we also introduced gene sets related to molecular functions from the MSigDB database. We selected 56 gene sets containing Hallmark and general gene sets from Joachimiak's research, which is publicly available at https://github.com/monarch-initiative/talisman-paper/tree/main/genesets/human.
Besides, we also derive seven gene sets from the study of sub-clonal evolution on gene expression in mouse B2905 melanoma cell lines, with the number of genes in each set ranging from 19 to 49. Three subclones to immunotherapy are identified from the B2905 melanoma cell line, i.e., high aggression and resistant (HA-R), high aggression and sensitive (HA-S), and low aggression and sensitive (LA-S). Then, EvoGeneX (2023) is used to identify adaptively up-regulated and down-regulated genes in each of HA-R, HA-S, and LA-S clades. Finally, the adaptively up- and down-regulated gene lists were subjected to the KEGG pathway enrichment analysis to obtain such seven gene sets. However, in our case study, we only utilized the seven gene sets analyzed from the clonal subline as the evaluation data of GeneAgent. We did not access or process any original data from clinical experiments.

Research involving human participants, their data, or biological material

Policy information about studies with human participants or human data. See also policy information about sex, gender (identity/presentation), and sexual orientation and race, ethnicity and racism.

Reporting on sex and gender	Not applicable.
Reporting on race, ethnicity, or other socially relevant groupings	Not applicable.
Population characteristics	Not applicable.
Recruitment	Not applicable.
Ethics oversight	Not applicable.

Note that full information on the approval of the study protocol must also be provided in the manuscript.

Field-specific reporting

Please select the one below that is the best fit for your research. If you are not sure, read the appropriate sections before making your selection.

☒ Life sciences ☐ Behavioural & social sciences ☐ Ecological, evolutionary & environmental sciences

For a reference copy of the document with all sections, see nature.com/documents/nr-reporting-summary-flat.pdf

Life sciences study design

All studies must disclose on these points even when the disclosure is negative.

Sample size	We conduct comparative experiments on 1106 gene sets from three distinct sources: literature curation (Gene Ontology), proteomics analysis (NeST system of human cancer proteins), and molecular functions (MSigDB). Specifically, to evaluate the recovery of literature-curated gene set functions, we used the same number of gene sets as those studied in the Hu et al.'s research (https://github.com/idekerlab/llm_evaluation_for_gene_set_interpretation/blob/main/data/), which contains 1000 terms randomly sampled from the GO Biological Process branch (GO-BP 2023-11-15 release). To evaluate the exploration of omics gene sets, we also collected the 50 gene sets provided in the Hu et al.'s research, where the genes encoding complexes of interacting proteins are identified by proteomic methods. The sample size of these evaluated gene sets is same as the data scales released in the original study.<br>Furthermore, we introduced 56 gene sets related to molecular functions from the MSigDB database, containing Hallmark and general gene sets from Joachimiak's research, which is publicly available at https://github.com/monarch-initiative/talisman-paper/tree/main/genesets/human. This sample size is over 75% (56/73) of the original data scales, containing all unique gene sets with convinced ground truth.<br>Besides, we derived 7 gene sets from the study of sub-clonal evolution on gene expression in mouse B2905 melanoma cell lines, with the number of genes in each set ranging from 19 to 49.
Data exclusions	Gene sets that only differed in “UP” and “DN” and those with unclear ground truth names were excluded from the MSigDB dataset.

Replication	All outputs of LLMs for evaluated gene sets are performed once based on the reproducible setting (temperature=0).<br>All experiment evaluations for the output of gene sets are carried out based on the fixed parameters.
Randomization	Random sampling is used to obtain the data in the previous study. Related original studies have elucidated the high quality of the sampled data. Therefore, we continue to use the same data or the subset in our study. The subset of data is selected by the researchers based on their domain knowledge. The proportion of subset of original data is determined to 75%-100%.
Blinding	In the evaluation of case study for testing 7 novel gene sets that are derived from the mouse B2905 melanoma cell lines, we invited two genomics experts to participate in the annotation for the results. They discerned and selected the superior response without knowing the method name of the output, substantiating their choice with pertinent comments. After synthesizing all comments, they are mandated to render a conclusive determination regarding which output best serves for users. Other assessments did not have a blinding setting.

Reporting for specific materials, systems and methods

We require information from authors about some types of materials, experimental systems and methods used in many studies. Here, indicate whether each material, system or method listed is relevant to your study. If you are not sure if a list item applies to your research, read the appropriate section before selecting a response.

Materials & experimental systems

n/a	Involved in the study
☒	☐ Antibodies
☒	☐ Eukaryotic cell lines
☒	☐ Palaeontology and archaeology
☒	☐ Animals and other organisms
☒	☐ Clinical data
☒	☐ Dual use research of concern
☒	☐ Plants

Methods

n/a	Involved in the study
☒	☐ ChIP-seq
☒	☐ Flow cytometry
☒	☐ MRI-based neuroimaging

Plants

Seed stocks	Not applicable.
Novel plant genotypes	Not applicable.
Authentication	Not applicable.
